# WJES and case reports/case series

**DOI:** 10.1186/1749-7922-2-11

**Published:** 2007-05-08

**Authors:** Luca Ansaloni, Fausto Catena, Ernest E Moore

## 

Case series (CS) and case reports (CR) consist either of collections of reports on the diagnosis and treatment of individual patients, or of a report on a single patient. Since its launch in March 2006, *World Journal of Emergency Surgery *(WJES) has received high numbers of CRs for publication: up until December 2006 they represented 39.2% of all articles submitted. Unfortunately the rejection rate of CRs in WJES, like many medical journals, is quite high, (85.2% of submitted CRs), and is significantly higher than other article types in the journal, where the rejection rate so far is 51.7%.

Since WJES is an electronic journal, whether or not a CR is published doesn't depend upon the available pages in the journal, but only upon the nature of the competing CRs. Ultimately, the low acceptance rate of CRs happens because, according to the principles of evidence based medicine, they provide a lower strength of evidence among clinical studies, being towards the base of the Evidence Pyramid [1], just above 'Expert Opinion' (Fig. 1).

**Figure 1:**
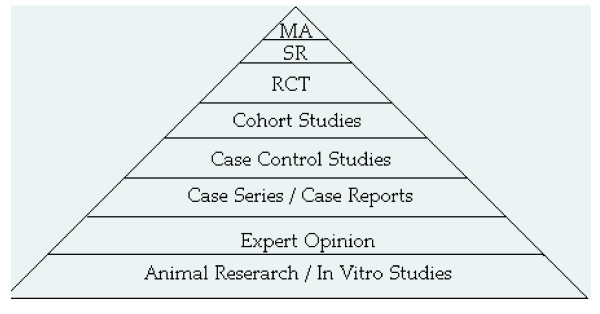
Evidence Pyramid (MA = metanalysis, SR = systematic review, RCT = randomised controlled trial, from  [1]).

On the other hand, observation and description has always been seen as the first step in science, and should be the same in clinical science. For this reason, since ancient times doctors have written CRs in a very "modern" manner by giving a clinical picture of the clinical case, followed by anatomic/physiological diagnosis, prognosis, and then discussion [2]. If the CR is intended as a way of discovering what is 'un-known' or 'unrecognized', it fits perfectly, as a scientific approach with the Karl Popper's hypothetico-deductive model [3]. CRs may be important sources of information about the care of patients because they describe important scientific observations missed or undetectable by "higher" clinical studies. These insights expand our knowledge and lead to new research, resulting in better and safer patient care [4,5]. Many important pathologies were discovered by clinicians who presented them to the world through a CR, for example, Burkitt's lymphoma [6], the acquired immune deficiency syndrome [7], and the new variant of Creutzfeldt-Jakob disease [8] were all brought to attention in this manner. Furthermore CRs are important for the detection of side effects of drugs, prompting some recent retractions from the market, such as weight reduction agents [9] and antihistamine drug [10]. In addition, in the fields of surgery and especially in emergency surgery to obtain the "evidence" that could be positioned at the top of the Evidence Pyramid is difficult and occasionally impossible to attain due to the large numbers required for the organization of efficacious randomised controlled trials, cohort and case-control studies.

With this editorial, having now clarified our point of view not only about the limits of CRs [11-13], but also their importance in evidence based medicine [14-18] we intend to lay out simple guidelines for acceptance criteria, content and format to allow CRs' publication in WJES.

As a first step we would like to ask authors to identify the reasons for publishing the CR in WJES. In the Appendix 1 we report the criteria for publishing CRs.

In Appendix 2 we describe the format with simple guidelines for writing up a CR. The CR should be structured into the following sections: Abstract, Background, Case Report and Discussion. Further details on each of these sections follows below.

## Abstract

CRs should start with an abstract of maximum of 350 words. The aim of the abstract is to allow readers to discern their levels of interest in the CR. The abstract should be structured into the same three sections as the main text in a succinct form: Background, Case Report and Discussion.

## Background

The background should convince the reader in a concise and relevant manner to continue reading and also provide all the necessary information about what the CR is about, providing its subject, purpose and value. This section should clarify why the CR is novel or merits publication with a brief description of the patient case and also a discussion of similar cases or studies in the context of a wider review of the literature on this topic. The literature review should list the strategy and coverage of the search and should include the database searched and the search terms used, providing enough elements for the reader to easily replicate the search.

## Case report

Within this section, the authors should provide the characteristics of the pathology, all significant data and interesting information about the patient and their lifestyle, which could be in some way linked to the condition. To respect patient privacy, it is important to omit all unessential personal information or data in order to anonymize the case. Informed consent is considered mandatory for publication and should be detailed at the end of this section. The CR should describe the patient's demographics and history, their laboratory and diagnostic data and the history of their medications. The case should be described in chronological order and with enough details to give the reader a chance to formulate their own opinion and evaluate the case's validity. It's important that a report stimulates inquiries, commentaries and remarks. Indeed, readers have the ability to, and are encouraged to post a comment on the published article, generating further discussion. Whilst the author should be succinct, and describe the case without leaving the reader doubtful about the correct management, they should not overload the reader with excess information. Fluency and clarity of the CR can be enhanced by the use of tables, graphs, figures and illustrations. Usually, most of the information contained in these additional parts should not be duplicated in the text. In particular colour pictures of histopathology, roentgenograms, electrocardiographs, and other diagnostic tests; skin manifestations; wounds; and other anatomical parts may be provided and add to the interest of the CR. It is imperative that any identifying features of a patient's photograph should be blocked out and patient permission for obtaining and using photographs must have been sought and included in the consent statement.

## Discussion

This is arguably the most important part of the article, because in this section the author should indicate the CR's accuracy, validity and uniqueness, comparing it with the published literature in order to derive new knowledge and applicability to practice. To obtain this the author must point out the value of the CR by demonstrating the validity of the diagnostic hypothesis and the therapeutic decisions, and comparing it with similar CRs if they exist. It is necessary to analyze the limits of the CR, describing the importance of each limit, but the main theme of the discussion should be the "lesson to be learned": if a CR doesn't teach anything new, it doesn't deserve to be published. Highlighting the practical applicability of the CR is important and should be clearly stated, relating to the eventual "evidence" already present in the literature and to the opportunities for future research. The discussion should conclude by briefly summarizing the CR, pointing out the lesson learned and joining it to eventual evidence based medicine recommendations.

We hope to have provided readers with a useful overview of the importance and structure of case reports for WJES, and welcome submissions of interesting and important cases to the journal.

## Appendix 1. Criteria for publishable case reports

Publishable CRs should meet one of the categories:

□ The first report of a new entity, for example, the first description of a disease, syndrome, diagnostic test, surgical procedure.

□ Additional examples that establish an entity from an isolated observation, such as the report of an already described, but rare (<5 cases already reported) or uncommon disease (10–15 cases already reported); the description of a rare, perplexing, or novel diagnostic features of a known disease, example of rare (<5 cases already reported) or uncommon (10–15 cases already reported), but not necessarily unexpected, behaviour in any disease.

□ Adverse events: the report of life-threatening adverse events, dangerous and predictable adverse effects that are poorly appreciated and rarely recognized in drugs or surgical procedures; the description of new medical errors or medication errors, rare or novel adverse drug reactions; the finding out of a device malfunction that results in patient harm; and the account of a therapeutic failure or a lack of therapeutic efficacy, clinical behaviour contrary to expectations based upon what we think we know.

□ A remarkably well-documented example of educational value (report therapeutic challenges, controversies, or dilemmas; teach humanistic lessons to the health care professional).

□ Other.

Specify:......................................................................................................................................................................................

## Appendix 2 – guidelines for writing patient case report manuscripts

1. Abstract

□ Structured.

□ Background.

□ Case report.

□ Discussion.

□ Not more than 350 words in length.

2. Background

□ Description of the subject matter.

□ Report of the purpose of the CR, its background information and pertinent definitions.

□ Details of the complete analytic literature review with its strategy search, using it to justify the merit of the CR.

□ Introduction of the patient case to the reader.

3. Case Report

□ Description of the case in a narrative form, providing patient demographics (age, sex, height, weight, occupation) and avoiding patient identifiers (date of birth, initials).

□ Description of the patient's clinical picture, listing his/her present illness, medical, family, social and medication history.

□ List of the patient's admission and throughout the case report pertinent findings on physical examination and laboratory values that support the case.

□ List of the diagnostic procedures that are pertinent and support the case.

□ Photographs of clinical findings, histopathology, roentgenograms, TC or RMN as they relate to the case, avoiding patient identifiers.

□ Patient's events in chronological order.

□ Description of the patient's medical and surgical treatments, with eventual side effects and complications.

□ Presence of enough detail for the reader to establish the case's validity.

□ Statement of achievement of a written consent from the patient for publishing the CR.

4. Discussion

□ Comparison of the case report with the literature review, describing similarities and differences between them.

□ List of the limits of the case report and description of its relevance.

□ Summary of the salient features of the case report.

□ Ascertainment of eventual causal and temporal relationship in the patient CR.

□ Justification of the eventual uniqueness of the CR.

□ Describe how the information learned applies to one's own practice.

□ List opportunities for research.

□ Indication of evidence-based recommendations and justified conclusion.
